# A risk prognostic model for patients with esophageal squamous cell carcinoma basing on cuproptosis and ferroptosis

**DOI:** 10.1007/s00432-023-05005-5

**Published:** 2023-07-05

**Authors:** Jianan Li, Jixuan Liu, Jixian Li, Alei Feng, Yuanliu Nie, Zhe Yang, Wentao Zhang

**Affiliations:** 1 Tumor Research and Therapy Center, Shandong Provincial Hospital, Shandong University, Jinan, 250021 Shandong People’s Republic of China; 2 Department of Pathology, Shandong Provincial Hospital, Jinan, 250021 Shandong People’s Republic of China

**Keywords:** Ferroptosis, Cuproptosis, Esophageal cancer, Clinicopathologic features, Tumor immune microenvironment

## Abstract

**Background:**

Cuproptosis, a form of copper-dependent programmed cell death recently presented by Tsvetkov et al., have been identified as a potential therapeutic target for refractory cancers and ferroptosis, a well-known form describing iron-dependent cell death. However, whether the crossing of cuproptosis-related genes and ferroptosis-related genes can introduce some new idea, thus being used as a novel clinical and therapeutic predictor in esophageal squamous cell carcinoma (ESCC) remains unknown.

**Methods:**

We collected ESCC patient data from the Gene Expression Omnibus and the Cancer Genome Atlas databases and used Gene Set Variation Analysis to score each sample based on cuproptosis and ferroptosis. We then performed weighted gene co-expression network analysis to identify cuproptosis and ferroptosis-related genes (CFRGs) and construct a ferroptosis and cuproptosis-related risk prognostic model, which we validated using a test group. We also investigated the relationship between the risk score and other molecular features, such as signaling pathways, immune infiltration, and mutation status.

**Results:**

Four CFRGs (MIDN, C15orf65, COMTD1 and RAP2B) were identified to construct our risk prognostic model. Patients were classified into low- and high-risk groups based on our risk prognostic model and the low-risk group showed significantly higher survival possibilities (*P* < 0.001). We used the “GO”, “cibersort” and “ESTIMATE” methods to the above-mentioned genes to estimate the relationship among the risk score, correlated pathways, immune infiltration, and tumor purity.

**Conclusion:**

We constructed a prognostic model using four CFRGs and demonstrated its potential clinical and therapeutic guidance value for ESCC patients.

**Supplementary Information:**

The online version contains supplementary material available at 10.1007/s00432-023-05005-5.

## Introduction

Esophageal squamous cell carcinoma (ESCC) is the sixth leading cause of cancer death and the eighth most common cancer in the world. It is a complex disease with a poor 5-year overall survival rate, partly due to late detection (Abnet et al. 2018). Endoscopic screening for ESCC is expensive and not effective enough in many high-risk regions (He et al. 2021). Thus, there is a need for a more convenient, accurate, and sensitive test based on biomarkers (Sheikh et al. 2023).

The tumor immune microenvironment (TIME) primarily consists of tumor cells, immune cells, and stromal cells that interact with the extracellular matrix and could directly or indirectly affect the occurrence and development of tumors (Li et al. 2022a).

Ferroptosis is a unique form of programmed cell death characterized by iron accumulation and lipid peroxidation, leading to the accumulation of reactive oxygen species (ROS) and cell death. Ferroptosis has been found to be associated with many diseases, including stroke, ischemia, cancer, and reperfusion-induced organ injury (Dixon et al. 2012; Yang and Stockwell 2008). Blocking ferroptosis has been found to be effective in treating reperfusion-induced organ injury and stroke in in vitro and in vivo models (Friedmann Angeli et al. 2014; Alim et al. 2019; Linkermann et al. 2014). Moreover, ferroptosis is considered to be a promising target for cancer therapy (Liang et al. 2020). According to Lu et al., cancer immunotherapy and TIME are also related to ferroptosis, which represents the most appealing approach for cancer therapy (Lu et al. 2021). Thus, we believe that a better understanding of the relationship between ferroptosis and TIME may lead to a more effective use of cancer therapy.

Copper is an essential nutrient for cell life in mitochondrial respiration, iron uptake, and many other antioxidant or detoxification processes, similar to other metals (Li et al. 2022b). Cuproptosis, a new form of cell apoptosis distinct from either of the programmed cell death that have been proposed previously, was introduced by Tsvetkov et al. (2022). Excessive copper leads to mitochondrial protein aggregation, resulting in the loss of iron-sulfur cluster proteins and ultimately causing proteotoxic stress and cell death (Cobine and Brady 2022). The relationship between copper and iron intake suggests the therapeutic potential between cuproptosis and ferroptosis, and this relationship has already been observed in hepatocellular carcinoma and colorectal cancer (Zhang et al. 2022; Li et al. 2022c). However, it is not yet known whether the crossing of cuproptosis-related genes (CRGs) and ferroptosis-related genes (FRGs) can be used as a predictor of prognosis and immune response in ESCC. In this study, we systematically analyzed the expression levels of cuproptosis- and ferroptosis-related genes (CFRGs) and their impact on the prognosis and TIME of ESCC patients. We hope that our study will contribute to the identification of viable predictive biomarkers of prognosis and immunotherapy response for ESCC patients.

## Materials and methods

### Data acquisition

The data of RNA sequencing (RNA-seq) and its corresponding clinicopathologic information of ESCC were downloaded from public databases, namely the Cancer Genome Atlas (TCGA) and Gene Expression Omnibus (GEO, https://cancergenome.nih.gov/, https://www.ncbi.nlm.nih.gov/geo/, GSE53624). The FRGs were obtained from the Molecular Signatures Database (http://www.gsea-msigdb.org/gsea/msigdb/), while the CRGs were obtained from previous publications by Tsvetkov et al. (2022). As the data were available online with usage allowance, additional ethical approval was unnecessary.

### CFRGs identification based on GSVA and WGCNA

To obtain the ferroptosis and cuproptosis scores of all samples, we conducted a GSVA analysis using the “GSVA” package in the TCGA (Tomczak et al. 2015) and GTEx database (Nangraj et al. 2020). We performed WGCNA and identified 4 optimal cuproptosis and ferroptosis-related genes (OCFRGs) based on the scores obtained above. To determine the optimum soft thresholding power, we performed the function “pickSoftThreshold” in R and found that it was 4. To analyze modules with different power, we calculated the topological overlap matrix (TOM) and diss TOM (1-TOM) to generate the gene dendrogram (Lin et al. 2020). Finally, we conducted a Pearson correlation coefficient analysis for the co-expression modules of cuproptosis-score and ferroptosis-score.

### Establishment and verification of a cuproptosis and ferroptosis-related gene prognostic prediction model

**Fig. 1:**
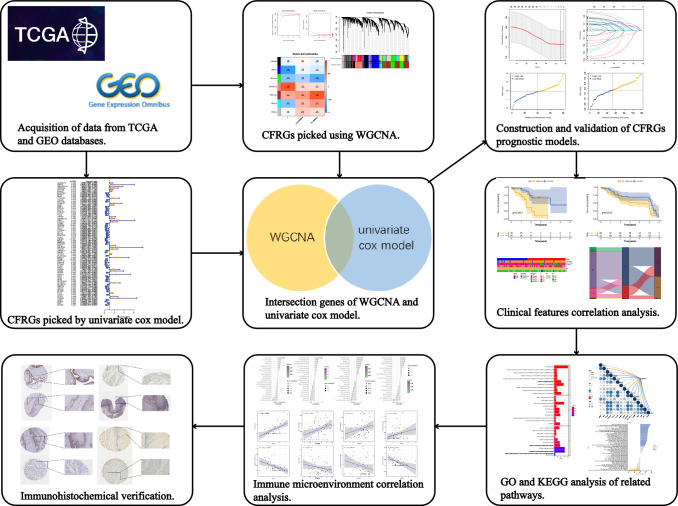
Workflow of the study. This figure shows the construction process and subsequent analysis of the CFRGs prognostic model

The detailed work process of our study is presented in Fig. 1. After screening out the CFRGs, we performed five machine learning methods, including Decision tree, extreme gradient boosting (XGBoost), gradient boosting decision tree (GBDT), random survival forest, and least absolute shrinkage and selection operator (LASSO), to estimate their prognostic value. Then, we performed univariate Cox regression and constructed a Lasso Cox model using the top-ranked 30 selected genes. The median risk score was considered the optimal cutoff value to divide all patients into two groups (high risk and low risk).

We evaluated the predictive performance of CFRGs using survival status distribution, principal component analysis (PCA), Kaplan–Meier (KM) analysis, and 1-, 3-, and 5-year receiver operating characteristic (ROC) curves, using the “survival”, "survminer," and "timeROC" R packages, respectively (Kong et al. 2020; Wang et al. 2020; Zhu et al. 2020). Finally, we used the GEO cohorts’ datasets as the external validation cohorts.

### Correlation analysis of clinical features and risk score

We conducted cox regression analysis via “ComplexHeatmap” to investigate the correlation between the CFRGs prognostic model and several clinicopathological features, including sex, gender, and TNM stage (Moreno-Ayala et al. 2021). To present the results intuitively, we used “ggalluvial” to estimate the relationship among survival status, risk score, and significant prognostic factors (Ma et al. 2022).

### Correlation analysis of Immune Infiltration and Risk Score

We calculated several prognosis-related tumor-infiltrating immune cells, including CD4 + T cells, CD8 + T cells, B cells, neutrophils, macrophages, and mast cells, in ESCC patients using algorithms such as “TIMER,” “CIBERSORT”, “CIBERSORT-ASB”, “QUANTISEQ”, “MCPCOUNTER”, “XCELL”, and “EPIC” (Lv et al. 2022). To further investigate the difference in immune infiltration level between two risk groups, we conducted single sample gene set enrichment analysis (ssGSEA) using the “ssGSEA” R package (Lv et al. 2022). We then used the “cibersort” algorithm to evaluate the Pearson correlation coefficient of the expression level of four OCFRGs with the immune cells, and the most significant immune cell and its R-value were calculated.

### Pathway enrichment analysis of GO

We conducted GO enrichment analyses between the two risk groups to further investigate the potential biological functions and pathways of our prognostic signature of CFRGs. Additionally, we collected several cuproptosis and ferroptosis-related pathways from previous publications, including Hippo, ErbB, Wnt, MAPK, NF-kB, PI3K/AKT, TGF-β, Notch, Ras, AMPK, JAK-STAT, TNF, PD-1/PD-L1, mTOR, and HIF-1 (Gao et al. 2014; Mizushima et al. 2002; Tian et al. 2006; Du et al. 2009; Naganuma et al. 2012; Qiu et al. 2014; Zhang et al. 2020).

Using GSVA enrichment analysis, we selected a hub gene set (https://www.gsea-msigdb.org/gsea/downloads.jsp) to conduct correlation analysis with the risk score, aiming to further investigate the correlation between the risk score and enrichment score. Additionally, we used the “GSVA” R package to estimate the active signaling pathway between the two risk groups. Finally, we calculated the differential expression levels of four CFRGs between ESCC tissue and its paired paracancerous samples.

### Mutation and tumor purity analysis

To evaluate the differences in mutation status between high and low risk subgroups, we used the MAFTOOL software to obtain their respective top mutational genes, types, and frequencies of mutations (Mayakonda et al. 2018). Based on the expression data, we obtained immune scores, stromal scores, and estimate scores via the “ESTIMATE” algorithm to assess the relationship between risk score and tumor purity (He et al. 2020). Additionally, we obtained the simple nucleotide variation data of ESCC patients from the TCGA database.

### Tissue samples and cell lines

For the in vitro experiments, we used three ESCC cell lines, including KYSE-150, KYSE-510, and EC9706, as well as the human normal esophageal epithelial cell line Het-1A. These cells were maintained in RPMI-1640 (Gibco, China) or DMEM (Gibco, China) supplemented with 10% fetal bovine serum (FBS, Excell Bio, Shanghai, China) in a humidified incubator at 37 °C with 5% CO_2_. KYSE-150, KYSE-510, and Het-1A cells were kindly donated by Dr. Xiangyan Liu, and EC9706 cells were purchased from Jennio Biotech, Guangzhou.

### Patients and specimens

We collected four frozen esophageal squamous cell carcinoma and its adjacent normal tissues from Shandong Provincial Hospital of Shandong University. Prior to surgery, all patients underwent gastroscopy, biopsy, and upper abdomen contrast-enhanced computed tomography (CT) to confirm the diagnosis of ESCC, and none of the patients received any anti-cancer treatment before surgery. Tissue specimens were preserved in RNA preservation solution and stored at − 80 °C until used. The study was conducted in accordance with the Declaration of Helsinki, and the protocol was approved by the Shandong Provincial Hospital (Shandong University) of China.

### Quantitative reverse transcription-polymerase chain reaction (qRT-PCR)

Total RNA from the cell lines, four ESCC tissue, and their paired paracancerous samples were isolated from tissues using the Accurate Biology kit (China). For cDNA, reverse transcription was performed using the TransScript uni all-in-one first-strand cDNA synthesis supermix for qPCR (TransGen Biotech, China), followed by quantitative reverse transcription-polymerase chain reaction (qRT-PCR) on cDNA using 2× Universal Blue SYBR Green qPCR Master Mix (Servicebio, China). The expression value of the target gene was normalized to that of the internal control gene GAPDH (CT GAPDH). All primers used in this research are listed in supplementary file 1.

### Immunohistochemistry (IHC)

In addition, we obtained four tissue microarrays (TMA) from Shanghai Outdo Biotech Company (Shanghai, China), with each TMA containing 30 esophageal squamous cell tumor tissues, paracancerous tissues, and distal normal tissues. We completed immunohistochemical staining with the help of Servicebio (Wuhan, China), using primary antibodies for immunohistochemistry including anti-MIDN (BB08169494), anti-COMTD1 (BB12065083), anti-RAP2B (BB12068902) from Bioss (China) and anti-C15orf65 (R98588) sourced from Novus Biologicals (USA). The TMA images were acquired using the TissueFaxs software (version 7.1.119) from Tissue-Gnostics^®^ (Vienna, Austria).

### Tissue‐microarray analysis

**Fig. 2:**
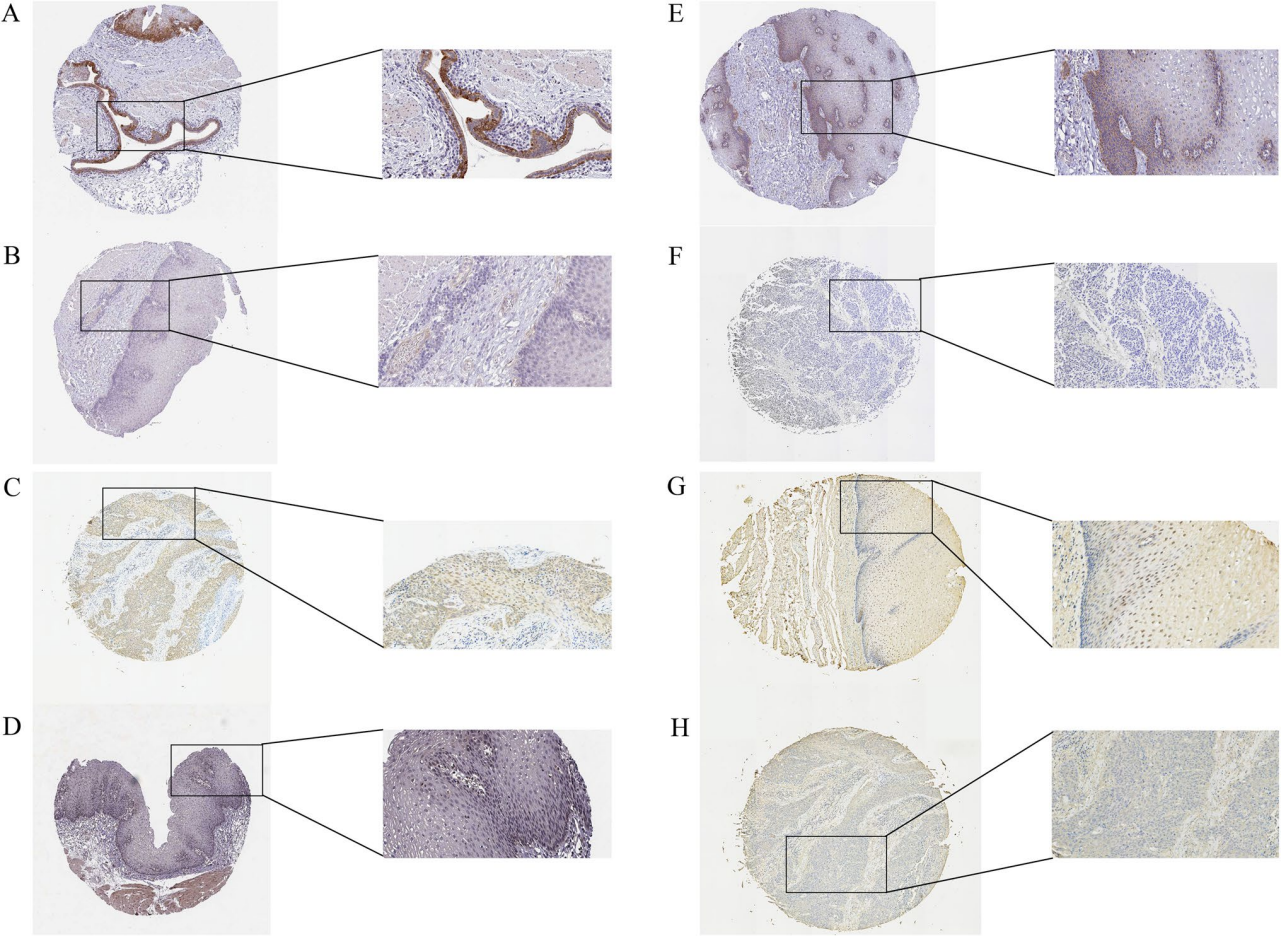
Mutation status and immunohistochemical verification. **A**, **B** Immunohistochemical findings for MIDN. **C**, **D** Immunohistochemical findings for C15orf65. **E**, **F** Immunohistochemical findings for COMTD1. **G**, **H** Immunohistochemical findings for RAP2B. Positive results are shown in the upper row and **A**, **B**, **D**, **E** was obtained from the Human Protein Atlas

The gene expression score for each sample was calculated by multiplying the percentage of stained cells and the staining intensity, expressed in arbitrary units (A.U.). Staining percentage was scored on a scale of 0–4, depending on the percentage of stained cells: 0 (0–5%), 1 (5–30%), 2 (30–50%), 3 (50–70%), and 4 (> 70%). Staining intensity was scored on a scale of 0 to 5, where 0 represents no staining, 1 represents moderate staining, and 5 represents high-intensity staining. Part of our scoring criteria is shown in Fig. 2A–H, and Fig. 2A, B, D, and E were obtained from the Human Protein Atlas.

### Statistical analysis

All experiments were repeated at least three times, and all the analyses were performed using R software (version 4.1.0). To filter out CFRGs, Pearson correlation coefficient analysis was utilized, and the Kaplan–Meier method was performed for univariate survival analysis (Parashar et al. 2019). The Wilcoxon test was used to evaluate expression levels, select differentially expressed genes (DEGs), estimate the proportion of tumor cells in the tumor tissue in high- and low-risk subgroups, and determine the difference in the proportion of somatic mutations. *P* values were exact, two-tailed, and values less than 0.05 were considered statistically significant.

## Results

### Clustering of CFRGs with prognostic value of clinical outcomes

**Fig. 3:**
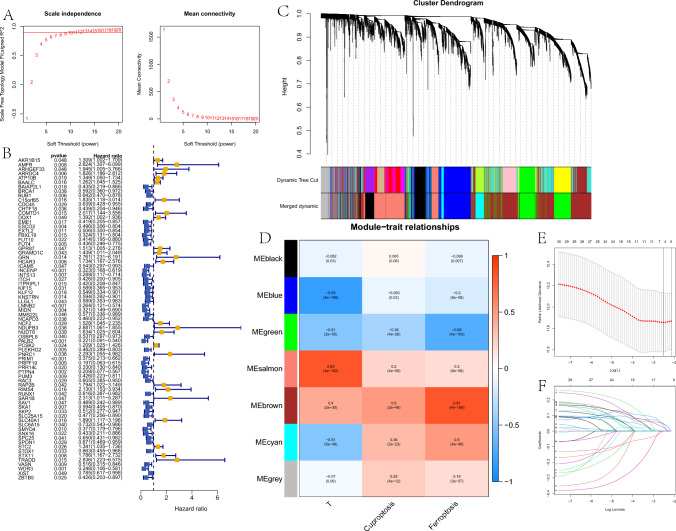
Screening of prognosis-related genes. **A** Scale-free fitting index for various soft-thresholding powers and analysis of the mean connectivity for different soft-thresholding powers. **B** Univariate Cox regression analysis of all CRFGs. **C** Hierarchical clustering-derived gene dendrogram. **D** The correlations among multiple modules, tumor, cuproptosis and ferroptosis. **E** Selection of the optimal penalty parameter for LASSO regression. **F** LASSO regression analysis

The soft threshold power was calculated using the pickSoftThreshold function in the WGCNA R package to select a soft threshold of 10 (Fig. 3A). Modules were determined using a dynamic cutting method, and each candidate module underwent cluster analysis using the “mergeCloseModules” function. Finally, a total of seven modules (represented by different colors) were identified using the Dynamic Tree Cut method. Next, we performed Pearson correlation analysis to investigate the correlation among co-expression modules, tumor size, cuproptosis score, and ferroptosis score. The “brown” module had the strongest correlation [*R* (Tumor) = 0.4, *R* (Cuproptosis) = 0.5, *R* (Ferroptosis) = 0.81, *P* < 0.001)] (Fig. 3D).

### Development of CFRGs prognostic model

To develop a predictive signature based on the cuproptosis score and ferroptosis score of ESCC patients, we performed univariate Cox analysis on genes in module “MEbrown” to identify survival-related CFRGs. The weights of every survival related CFRG were evaluated using five machine learning algorithms, including random forest, decision tree, LASSO, XGBoost, and GBDT. We then selected the top 30 CFRGs to screen genes and build a prognostic model using LASSO COX regression analysis. Four genes were chosen for the establishment of the CFRG prognostic model based on the optimal setting for the tuning parameter *λ*, among which C15orf65, COMTD1, and RAP2B were considered to be causative or risk factors, and MIDN was indicated as a protective factor. The risk score was calculated using the following formula

$${\text{Risk Score}} = \left( {{-}0.59198 \times {\text{ MIDN exp}}.} \right) + \left( {0.34205 \times {\text{ C}}15{\text{orf}}65{\text{ exp}}.} \right) + \left( {0.26762 \times {\text{ COMTD}}1{\text{ exp}}.} \right) + \left( {0.30334 \times {\text{ RAP}}2{\text{B exp}}.} \right).$$

**Fig. 4:**
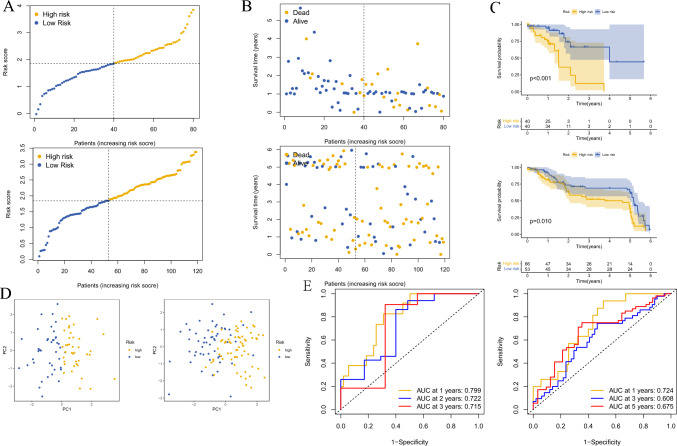
Construction of risk prognostic model. **A** The distribution and median value of the risk scores in the training cohort and the test cohort. **B** The distributions of OS status and risk score in the training cohort and the test cohort. **C** Kaplan–Meier curves for the OS of patients in the high-risk group and low-risk group in the training cohort and the test cohort. **D** The PCA plot of the training cohort and the test cohort. **E** AUC of 1, 3, and 5 year ROC curves verified the prognostic performance of the risk score in the training cohort and the test cohort

Independent validation datasets were obtained from the GEO database (http://www.ncbi.nlm.nih.gov/geo/), and patients were stratified into a high-risk group and a low-risk group based on the median of their risk scores in the training cohort and analyzed (Fig. 4A). The risk score distribution, gene expression, and OS status of patients in the training and validation cohorts are shown in Fig. 4B. PCA analysis indicated that different risk groups were distributed in two directions (Fig. 4D). After performing KM analysis, we found that the OS was significantly different (*P* < 0.001) between high- and low-risk subgroups in both TCGA and GEO cohorts (Fig. 4C). ROC curve analysis was performed to assess the sensitivity and specificity of the risk model, and the area under the ROC curve (AUC) was 0.799 for 1 year, 0.722 for 3 years, and 0.715 for 5 years in the training set (Fig. 4E) and 0.724 for 1 year, 0.608 for 3 years, and 0.675 for 5 years in the validation set (Fig. 4E).

### Clinical features and differential expression of genes

**Fig. 5:**
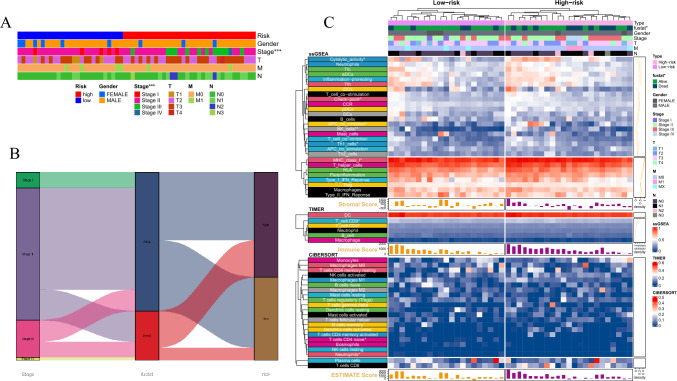
Clinical features and differential expression analysis. **A** Differences in clinicopathologic features and expression levels of CFRGs between the high-and low-risk group. **B** Sankey diagram of TNM stage in groups with different GSVA scores and survival outcomes. The asterisks represented the statistical *P* value (*P < 0.05; **P < 0.01; ***P < 0.001). **C** The correlation among clinical features, immune infiltrating, tumor purity and risk score

To examine the different clinical characteristics between two groups, sex, gender, and TNM stage were included as covariates, and the correlations did not reach significance except for tumor staging (Fig. 5A). Using the Sankey diagram, we could unambiguously assess that patients in the high-risk group experience a larger share of death, and the most associated clinical factor with death is tumor staging (Fig. 5B). To facilitate a better comprehension of the subject matter, we have prepared a comprehensive heatmap (Fig. 5C) that delineates the intricate interplay between tumor purity, immune infiltration, and clinical features.

### Prediction of immune infiltration of CFRGs in tumors

**Fig. 6:**
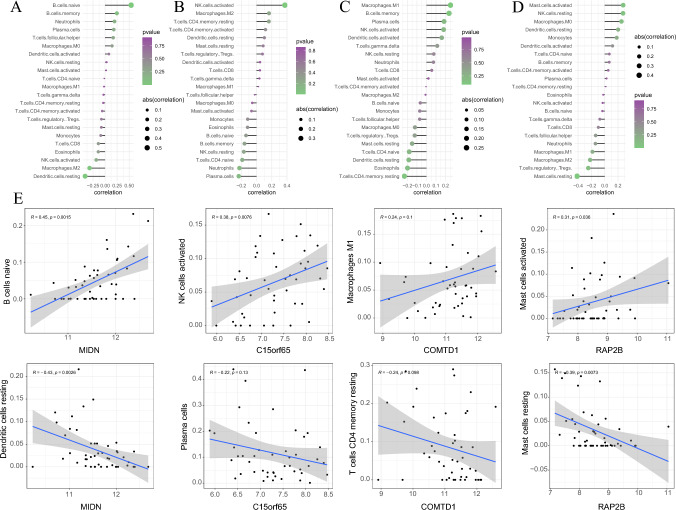
Immune cell infiltration of OCFRGs. **A**–**D** The relation between immune infiltration and four OCFRGs expression. **E** The most associated infiltrating immune cells with different OCFRGs

To further investigate the relationship between infiltrating immune cells and gene expression levels, we used the “CIBERSORT” algorithm and found that MIDN expression was significantly positively correlated with B cells naive, and C15orf65 expression was negatively correlated with plasma cells, while both genes were positively correlated with antigen-presenting cells. Regarding the two other genes, COMTD1 expression was linearly related to macrophages M1, and RAP2B showed a positive correlation with activated mast cells. The detailed correlation between gene expression and immune cell infiltration based on CIBERSORT is listed in Fig. 6.

### CFRGs-related signaling pathways in tumors

**Fig. 7:**
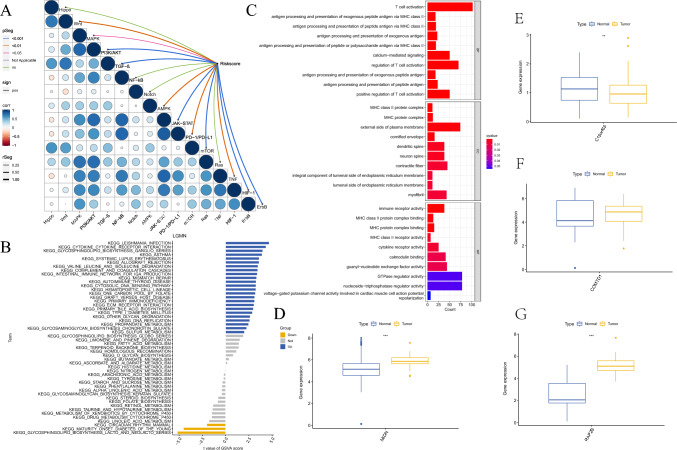
Pathway enrichment analysis of the cuproptosis-related genes. **A** Correlation analysis of biological processes and risk scores. **B** Different biological processes between high and low risk groups. **C** Enriched items in gene ontology analysis and Kyoto Encyclopedia of Genes and Genomes. *BP* biological process, *CC* cellular component, *MF* molecular function. **D**–**G** Expression of 4 different OCFRGs in esophageal squamous cell carcinoma (ESCC) patients’ tumor tissues and paracancerous tissues

Gene ontology (GO) was used to enrich the correlated biological process, cellular component, and molecular function. The results showed that the enrichment of DEGs mainly presented in T cell activation, calcium-mediated signaling, and antigen processing and presentation pathways. The most related cellular components were MHC class II protein complex, MHC protein complex, and the external side of the plasma membrane. Furthermore, for molecular function, immune receptor activity, MHC class II protein complex binding, and MHC protein complex binding showed evidence of strong positive correlation (Fig. 7C). GSVA enrichment focused primarily on several important pathways, and the relationship between these pathways and the risk score was displayed in Fig. 7A. We found that the Wnt, MAPK, PI3K/AKT, TGF-β, AMPK, JAK-STAT, PD-1/PD–L1, mTOR, TNF, HIF-1, and ErbB pathways presented a positive correlation with the risk score (Fig. 7A), and Wnt, PI3K/AKT, TGF-β, AMPK, JAK-STAT, PD-1/PD-L1, mTOR, TNF, HIF-1, and ErbB showed strong correlations. Regarding the different activated pathways between high- and low-risk groups, we performed GSVA to enrich the activated pathways between the two subgroups and found that cytokine receptor interaction, intestinal immune network for IGA production, and complement and coagulation cascades were significantly enriched (Fig. 7B). The Wilcoxon rank-sum test was used to evaluate expression levels, and we found that MIDN, COMTD1, and RAP2B were highly expressed in tumor tissues and lowly expressed in normal tissues. However, the difference in COMTD1 expression did not reach significance. For C15orf65, we observed the opposite tendency (Fig. 7D–G). We observed a similar tendency in the qRT-PCR and IHC experiments. Files for qRT-PCR and IHC are available in supplementary files 2 and 3.

### Tumor immune microenvironment analysis

**Fig. 8:**
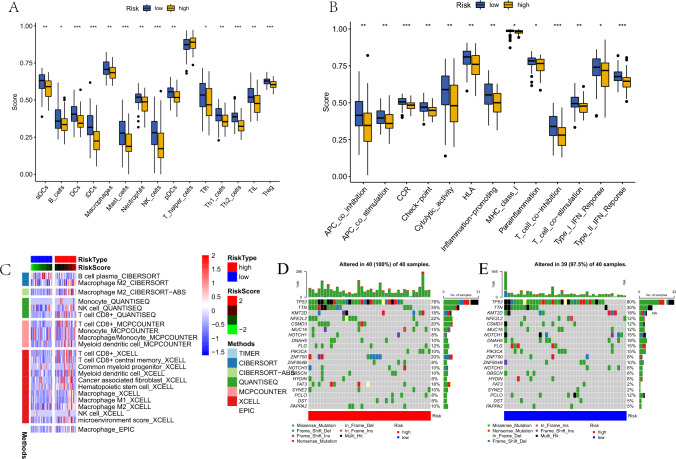
Immune Infiltration and Tumor Purity Analysis. **A** The different immune infiltration between high and low‐risk groups. **B** The immune‐related function between high and low‐risk groups. **C** The level of immune cell infiltration between high and low‐risk groups using different methods. **D** The mutation status in the high-risk score group in Esophageal squamous cell carcinoma (ESCC) patients. **E** The mutation status in the low-risk score group in ESCC patients

With respect to the tumor immune microenvironment, we found that in the high-risk subgroup, activated dendritic cells (aDCs), B cells, dendritic cells (DCs), immature dendritic cells (iDCs), macrophages, mast cells, neutrophils, NK cells, plasmacytoid dendritic cells (pDCs), follicular helper T cells, Th1 cells, Th2 cells, tumor-infiltrating lymphocytes (TILs), and regulatory cells (Tregs) had significantly higher infiltrating abundance (Fig. 8A). In terms of immune-related functions between the high- and low-risk groups, several important functions were significantly different, such as T cell co-inhibition, T cell co-stimulation, and checkpoint functions (Fig. 8B).

### Mutation status and tumor purity analysis

To further explore the function of our established model, we analyzed the mutation status of the high- and low-risk groups in the TCGA cohort. We found that TP53, TNN, KMT2D, and NFE2L2 were the most frequently mutated genes, and regardless of the mutated genes, missense mutation was the most common mutation type. The detailed relationship among mutation type, number of samples, and TMB in the high- and low-risk groups is displayed in Fig. 8D, E.

Tumor purity is composed of the immune score and estimate score. To analyze the relationship between the risk score and tumor purity, we used the “ESTIMATE” algorithm to obtain the immune score, stromal score, and estimate score and assessed the proportion of tumor cells in the tumor tissue in the high- and low-risk subgroups using the Wilcoxon rank-sum test. Immune score (*R* = 0.39), estimate score (*R* = 0.33), and stromal score (*R* = 0.22) were all positively correlated with the risk score and showed significant differences.

## Discussion

ESCC is a highly heterogeneous tumor, and its molecular characteristics and unique tumor microenvironment greatly affect the final efficacy. Despite diagnostic and therapeutic advances that have made ESCC more detectable, the prognosis remains poor, and a more accurate prognostic model is still needed. Cuproptosis, a novel form of copper-dependent programmed cell death, has already been developed as a diagnostic marker or a promising treatment in a number of solid tumors (Song et al. 2022; Bao et al. 2022). According to Tsvetkov et al. (2022), copper ionophore therapy is more effective for tumors undergoing mitochondrial respiration (Zheng et al. 2022). Therefore, further exploration of the association between cuproptosis and ESCC is desirable and may provide a new diagnostic and therapeutic strategy for ESCC.

Here, we not only developed a cuproptosis and ferroptosis-related signature but also evaluated changes in the integrative expression of CFRGs in ESCC patients at both genetic and transcriptional regulatory levels. First, we obtained DEGs of cuproptosis and ferroptosis from previous literature (Dixon et al. 2012; Tsvetkov et al. 2022). After consensus clustering assessment, we conducted univariate Cox regression and LASSO analyses on these CFRGs to determine their association with the prognosis of ESCC. Then, a prognostic risk model was built, and the formula for the risk score was obtained based on four hub genes (MIDN, C15orf65, COMTD1, RAP2B). According to the median score, patients were then divided into two groups, and differences between the two groups in several clinical prognostic features, immune infiltration, signaling pathways, and tumor purity were noted.

Of note, the two groups had significantly different immune infiltration features and activated tumor signaling pathways. The low expression group exhibited poorer immune infiltration levels, especially in some prognosis-related immune infiltrating cells, such as dendritic cells (DCs), interdigitating dendritic cells (iDCs), macrophages, and mast cells. According to Teixeira Farinha et al. (2021), Wang et al. (2020), better immune infiltration may indicate a better response to immune checkpoint inhibitors (ICIs), leading to better overall survival. Given the strong correlation between prognosis and immune infiltration, we further analyzed the relationship between the expression level of the four hub genes and immune infiltration. Kam et al. (2022) reported that high densities of peritumoral proliferating B cells are related to improved prognostic significance in ESCC, which confirms from another perspective that higher expression of MIDN may be associated with improved survival. As for NK cells, an innate immune cell with potent cytolytic activity against tumors, they have already been reported to be associated with a favorable outcome in ESCC (Baba et al. 2020); however, this is the first time that the expression of C15orf65 is noted to be related to the prognosis of ESCC and the infiltration of NK cells. Further, M1 macrophages have already been evidenced to be involved in inhibiting ESCC cell migration and invasion and the density of M1 macrophages is negatively correlated with tumor T staging, which could serve as a favorable prognostic factor in ESCC patients (Wang et al. 2013; Elpek et al. 2001). Notably, we first found that the expression levels of RAP2B may be related to mast cell activation. Additionally, our study is the first to demonstrate that the expression levels of RAP2B may be related to mast cell activation, and the density of mast cells in esophageal muscularis propria was confirmed to be associated with tumor invasion and served as a predictor of favorable survival for ESCC patients.

The expression data and bioinformatics analysis suggest that the expression of MIDN, C15orf65, and RAP2B is upregulated, whereas the expression of COMTD1 is downregulated in ESCC compared to normal tissues. This finding was also confirmed in clinical surgical specimens by PCR and IHC. MIDN, which regulates parkin expression, has been associated with Parkinson's Disease by regulating neurite outgrowth and Parkin expression in neuronal cells (Obara et al. 2019). This study is the first to report on the association between MIDN and ESCC. C15orf65 is a gene whose association with cancer prognosis is reported for the first time, and further studies are needed. COMTD1, which is known to be associated with cardiac hypertrophy (Pfleger et al. 2020), is first discovered to be related to the prognosis of ESCC. Previous publications have revealed that RAP2B might be related to an advanced tumor stage and grade in ESCC, while no study has been conducted to demonstrate the expression and function of other CFRGs in ESCC. Moreover, RAP2B is already found to be implicated in numerous biological processes such as platelet activation, autophagy, and signal transduction (Canobbio et al. 2008; Mansilla Pareja et al. 2019; Gao et al. 2021), and its association with proliferation, migration, and invasion has been reported in various cancers such as breast cancer, cervical cancer and bladder cancer (Staalesen et al. 2004; Li et al. 2018; Zhang et al. 2016). GO and KEGG analyses revealed that these CFRGs were enriched in T cell activation and antigen processing, and the dysregulation of these signaling pathways is shown to be implicated in the progression of ESCC.

However, this study has some limitations. The analysis data are all from public databases, and its retrospective nature is limited by an inherent case selection bias. Further studies in vivo are necessary to verify the accuracy of our prognostic model.

## Conclusion

We conducted a comprehensive analysis of the profiles of copper- and iron-regulating genes (CFRGs) in esophageal squamous cell carcinoma (ESCC) and identified a diagnostic and therapeutic signature related to cuproptosis and ferroptosis for ESCC patients. This signature comprises four CFRGs: MIDN, C15orf65, COMTD1 and RAP2B. To gain a better understanding of the specific roles of these CFRGs in ESCC, we analyzed their clinical features, immune infiltration, tumor purity, and tumor immune microenvironment (TIME). Our study demonstrates the potential clinical implications of CFRGs, suggesting that cuproptosis and ferroptosis may be promising targets for the treatment of ESCC patients.

## Supplementary Information

Below is the link to the electronic supplementary material.

Supplementary file1 (DOCX 42 KB)

Supplementary file2 (RAR 2418 KB)

Supplementary file3 (DOCX 41 KB)

## Data Availability

Data are available in a public, open access repository. Data are available upon reasonable request. All data relevant to the study are included in the article or uploaded as supplementary information.
