# Supplementary material for: A risk prognostic model for patients with esophageal squamous cell carcinoma basing on cuproptosis and ferroptosis
Source: J Cancer Res Clin Oncol. 2023 Jul 5;149(13):11647–59. doi: 10.1007/s00432-023-05005-5 (PMC10465684; doi:10.1007/s00432-023-05005-5)
Supplement: Supplementary file 1 — Supplementary file1 (DOCX 42 KB) [file 432_2023_5005_MOESM1_ESM.docx]

A Risk Prognostic Model for Patients with Esophageal Squamous Cell Carcinoma basing on Cuproptosis and Ferroptosis

Running title: A prognostic model for ESCC

Jianan li^1^, Bangqi Ji^2^, Liyan Zhang^3^, Yuanliu Nie^1^, Jixian Li^1^, Zhe Yang^1*^ and Wentao Zhang^1*^

^1^Tumor Research and Therapy Center, Shandong Provincial Hospital, Shandong University, Jinan, Shandong, 250021, People’s Republic of China

^2^Department of Imaging, Shandong Rehabilitation Hospital

^3^Department of Ultrasound,Sunshine Union Hospital

*** Correspondence:**Zhe Yang and Wentao Zhang
[sdslyyyz@sina.com](mailto:sdslyyyz@sina.com); [wentaozhang9683@gmail.com](mailto:wentaozhang9683@gmail.com)

Number of words: 5865

Number of tables/figures: 7

Number of supplementary files: 4

# Supplementary Data

The included primer sequence used in our research.

| **Primers’ name** | **The sequences of primers** | |
| --- | --- | --- |
| Homo-MIDN-F | CCTCATGTCTCAGGCCTCAAG |  |
| Homo-MIDN-R | GTGCCACGGACGCTTAAATAAA |  |
| Homo-C15orf65-F | CTGATCCACCCAGCTATTCATCT |  |
| Homo-C15orf65-R | CAAGGAGGCAGTTGTTCAGATT |  |
| Homo-COMTD1-F | GAACTGCTCCGCCTACTACGA |  |
| Homo-COMTD1-R | CGTTCGTTTAGGTTTCGCACAC |  |
| Homo-RAP2B-F | AGCATGTACGTTTCTCCCTGATT |  |
| Homo-RAP2B-R | TTTCCACCTTCTCTCCCTCCTTT |  |
