# Supplementary material for: A risk prognostic model for patients with esophageal squamous cell carcinoma basing on cuproptosis and ferroptosis
Source: J Cancer Res Clin Oncol. 2023 Jul 5;149(13):11647–59. doi: 10.1007/s00432-023-05005-5 (PMC10465684; doi:10.1007/s00432-023-05005-5)
Supplement: Supplementary file 3 — Supplementary file3 (DOCX 41 KB) [file 432_2023_5005_MOESM3_ESM.docx]

Supplementary Material

A Risk Prognostic Model for Patients with Esophageal Squamous Cell Carcinoma basing on Cuproptosis and Ferroptosis

Running title: A prognostic model for ESCC

Jianan li^1^, Bangqi Ji^2^, Liyan Zhang^3^, Yuanliu Nie^1^, Jixian Li^1^, Zhe Yang^1*^ and Wentao Zhang^1*^

^1^Tumor Research and Therapy Center, Shandong Provincial Hospital, Shandong University, Jinan, Shandong, 250021, People’s Republic of China

^2^Department of Imaging, Shandong Rehabilitation Hospital

^3^Department of Ultrasound,Sunshine Union Hospital

*** Correspondence:**Zhe Yang and Wentao Zhang
[sdslyyyz@sina.com](mailto:sdslyyyz@sina.com); [wentaozhang9683@gmail.com](mailto:wentaozhang9683@gmail.com)

Number of words: 5865

Number of tables/figures: 7

Number of supplementary files: 4

# Supplementary Figure

This is the original microscopy images and original files of our tissue microarrays obtained by TissueFaxs software. The [sketch map](javascript:;) shows the position of our tissue.

Due to the unwieldy size of the files, we were unable to upload them via suitable means. In the event of review, we will provide supplemental materials through electronic cloud platforms, and apologize for any additional workload this may cause.
